# 

**Published:** 1882-10

**Authors:** 


					﻿ESTABLISHED IN 1855.
Old Serie*.
Vol. XX-No. 7.
OCTOBER, 1882.
New Series
Vol. Il-No. I
ATLANTA
MEDICAL REGISTER.
(ATLANTA MEDICAL ANI) SURGICAL JOURNAL.}
EDITED ET
JAMES B. BAIRD, M.D.
H. H. DICKSON. PUBLISHER. TERMS. $2.50 A YEAR IN ADVANCE.
ATLANTA, GEORGIA;
H. II. DICKSON, BOOK AND JOB PRINTER AND BOOKBINDER.
1882.
				

